# The efficacy and safety of itopride in feeding intolerance of critically ill patients receiving enteral nutrition: a randomized, double-blind study

**DOI:** 10.1186/s12876-021-01712-w

**Published:** 2021-03-19

**Authors:** Eman Mohamed Elmokadem, Radwa Maher EL Borolossy, Ahmed M. Bassiouny, Maha Gamil Hanna, Ebtissam Abdel Ghaffar Darweesh, Nagwa A. Sabri

**Affiliations:** 1 Faculty of Pharmaceutical Sciences and Pharmaceutical Industries, Future University in Egypt, Cairo, Egypt; 2 Faculty of Pharmacy, Ain Shams University, Cairo, Egypt; 3 Faculty of Medicine, Ain Shams University, Cairo, Egypt; 4 Faculty of Medicine, Cairo University, Giza, Egypt

**Keywords:** Itopride, Metoclopramide, Enteral feeding intolerance, Gastric residual volume, Ultrasonography

## Abstract

**Background:**

Enteral feeding intolerance (EFI) is a frequent problem in the Intensive care unit (ICU) and is associated with poor clinical outcomes leading to worse prognosis in terms of mortality and ICU stay. Nowadays, prokinetic drugs are the mainstay of therapy in EFI. However, available prokinetics have uncertain efficacy and safety profiles. Itopride, is a prokinetic agent which is different and unique from the available prokinetics because of its dual mode of action as well as its tolerability and safety. The current study compared the efficacy and safety of Itopride against metoclopramide for EFI in critically ill patients. Moreover, it tested the utility and applicability of ultrasonography to measure gastric residual volume (GRV) in this population.

**Methods:**

This randomized, double-blind study included 76 EFI patients who were randomly assigned to either Itopride or metoclopramide group. The primary outcome was to measure GRV by ultrasonography. Secondary outcomes included the percentage ratio of enteral feed volume, energy and protein received by patients over 7 days of treatment, ICU length of stay, safety parameters and occurrence of infectious complications or vomiting.

**Results:**

Thirty-five patients of each group completed the study. At day 7, itopride significantly decreased GRV compared with metoclopramide group (*p* = 0.001). Moreover, there was a significant increase in the ratios of received enteral nutrition feed volume, calories, and protein after the one-week therapy in the itopride group more than the metoclopramide group (*p* = 0.001), (*p* = 0.002), (*p* = 0.01), respectively and there were no differences in any secondary outcomes or adverse events between the two groups.

**Conclusion:**

In critically ill patients with EFI, itopride was well tolerated with superior efficacy to metoclopramide. In addition, we demonstrated that ultrasonography is a simple, non-invasive, inexpensive, and undemanding method for GRV measurements and can offer reliable assessments in the gastric emptying modality.

**Trial registration:**

The trial was registered in ClinicalTrials.gov (NCT03698292). Date: October 5, 2018

## Background

Nutritional support for critically ill is now recognized as an integral part of patient care [1, 2]. Enteral nutrition (EN) has been the preferred means of nutritional support for feeding critically ill patients because of its favorable morbidity effects**,** lower cost, enhancement of gut immune function and its association with less septic complications compared to parenteral nutrition [3, 4].

However, delivery of adequate EN might be prohibited by critical illness induced gastrointestinal (GI) dysmotility resulting in elevated gastric residual volumes (GRVs). Many of the conditions associated with admission to the intensive care unit (ICU) cause delayed gastric emptying and result in GI dysfunction such as multi trauma, hyperglycemia, burns, mechanical ventilation, cardiac surgery, renal dysfunction, respiratory failure, medications, or the disease process itself. In critically ill patients, when gastric emptying was assessed, almost half of them showed delay in gastric emptying consistent with enteral feeding intolerance (EFI) [5].

EFI, defined as the failure to provide sufficient EN to critically ill patients due to delay of gastric emptying with the absence of mechanical blocking, is a common problem in critically ill patients with a stated prevalence of 30–46% and is accompanied by cumulative energy deficit, prolonged ICU stays, decreased ventilator-free days, and increased mortality [5, 6].

Accordingly, there is significant interest in therapies that enhance gastric motility and can alleviate feeding intolerance**.** Recent guidelines for the assessment and provision of nutritional support therapy in the adult critically ill patients have proven the significance of using prokinetic drugs to enhance gastric feeding tolerance and consequently improve clinical outcomes [3, 7].

Prokinetic agents such as metoclopramide, erythromycin, cisapride, and domeperidone have been used to enhance gastric emptying and are commonly used in the ICUs [8]. The safety profile of available prokinetic agents is a major concern when selecting therapies for EFI treatment. Cisapride is associated with QT interval prolongation in the electrocardiogram (ECG), and infrequent but serious cardiac arrythmias [9], while domeperidone has been described to cause gynecomastia and galactorrhea [10]. Erythromycin was associated with QT prolongation, multidrug resistant organisms' super-infection and drug-drug interactions [11].

Metoclopramide is the most commonly used prokinetic drug to overcome delayed gastric emptying, However, it is correlated with central nervous system (CNS) adverse drug effects (ADEs) and QT prolongation [12]. Moreover, the effects of the drug decrease rapidly with time where after a few days of treatment with metoclopramide, tachyphylaxis occurs such that success of feeding is less than 20% by day 3 of therapy in patients with high GRVs [13].

Thus, in view of these safety concerns with the currently available prokinetic agents, this has led to the search for another prokinetic agent with equivalent or better effectiveness, favorable tolerability profile and lower side effect potential.

Itopride hydrochloride, a prokinetic drug that has been reported to enhance GI motility through a dual mode of action; by preventing the effect of dopamine on the D2 receptors of the cholinergic nerves in the post-synaptic region. It also prevents the hydrolysis of the acetylcholine by the enzyme acetyl cholinesterase and thereby promotes GI motility [14, 15]. Since itopride does not cross the blood brain barrier thus does not exhibit ADEs related to CNS, it also hardly increases prolactin levels and does not prolong the QT interval [16].

Although GRV measurement provide a useful clinical endpoint in prokinetic drug studies, however, the current diagnostic tools for evaluating gastric emptying (GE) which include: scintigraphy, which is considered the “gold standard” for GE evaluation, the wireless motility capsule test, GE breath test, magnetic resonance imaging, fluoroscopy, and paracetamol or synthetic glucose absorption, all these methods have technical limitations, are expensive, complex, or not readily available in the ICU setting making the evaluation of GE a challenging task in clinical practice [17].

Ultrasonography (USG) is a non-invasive, inexpensive diagnostic test with a good inter-observer agreement compared to scintigraphy, and which provides real-time structural and functional information regarding most parameters of gastric motility. It doesn't require radiation and can be carried out at the bed side [18, 19].

In view of the aforementioned trends and knowing that to date, minimal data are available to support the clinical efficacy of Itopride in critically ill patients. Therefore, we conducted a clinical study to determine the efficacy and safety of Itopride in critically ill patients with EFI in comparison with metoclopramide. In addition, it tested the utility and applicability of USG to measure GRV in this population.

## Methods

### Study design and setting

This was a prospective randomized, double blind, comparator-controlled, study conducted between October 2018 and December 2019 at the ICU of Alkasr Al -Aini hospital, Cairo, Egypt.

### Ethical considerations

The ethical approval for both the scientific and the ethical aspects to conduct the study was obtained before initiation of the study from the committee of Ethics of Faculty of Pharmacy, Ain Shams University, and the research ethics committee for experimental and clinical studies at faculty of Pharmaceutical Sciences and Pharmaceutical Industries, Future University, Cairo, Egypt.

Patients or their legal guardians signed informed consent for inclusion in this study. All study procedures followed the Helsinki Declaration for protecting human subjects and compiled with Good Clinical Practice Guidelines. The trial was registered in ClinicalTrials.gov (NCT03698292).

Without prejudice, a patient can withdraw from the study at any time. For whatever reason, the physician could discontinue the participation of any patient, including inability to comply with the protocol. If a patient withdrew from the study or was withdrawn from it, this was noted on the case report form along with the reason for withdrawal.

## Methodology

### Study population

Patients were eligible to participate in the study, if they were admitted to the ICU and were expected to stay in it for at least one week and fulfilled the inclusion criteria which was: Age between 18 and 60 years of both genders who were prescribed enteral feeding and diagnosed with EFI which was determined by the following criteria: a 4-h GRV measurement by USG; and a GRV of ≥ 250 ml on one or more of the measurements or the development of vomiting, regurgitation, abdominal pain, or abdominal distention during enteral feeding.

The study excluded the following patients: A. Use of any prokinetic within 48 h before participating in the study. B. Known hypersensitivity to itopride or metoclopramide. C. Hemodynamic instability or occurrence of cardiac arrhythmia or prolonged QT interval of > 480 ms on a 12-lead ECG. D. Acute CNS infection diagnosis or severe brain injury. E. GI surgery ≤ 6 weeks before enrollment in the study, suspicious GI obstruction, hemorrhage, perforation, or history of GI disease, history of total/partial gastrectomy or esophagectomy, F. Extubation is expected within 48 h. G. weight > 150 kg, pregnancy or clinically significant renal or hepatic dysfunction.

### Study intervention

Eligible patients fulfilling the inclusion criteria were randomly assigned, using a computer random number generator program (Stattrek.com/statistics/random-number-generator), where a random list of numbers for patient allocation was produced and participant randomization assignment remained concealed in sealed envelopes. The study was double blinded; patients, clinicians, radiologist, and unit staff responsible for assessments remained blind from randomization. Study medications were prepared by an unblinded pharmacist to ensure correct treatment assignment however, this pharmacist was not aware of the study objectives nor involved in outcome assessment or other care. Standard operating procedures assured that all other operational personnel remained blinded to treatment assignment. Patients were assigned to one of the two groups as follows:

1. *Itopride group* These patients received 50 mg Itopride (Ganaton ® Kahira Pharmaceuticals Co., Cairo Egypt Under license from Abbott Laboratories) enterally t.i.d.
2. *Metoclopramide group* These patients received 10 mg metoclopramide (Primperan ® Sanofi Aventis) intravenously every 6–8 h. The treatment duration was 7 days for both groups.

*Enteral Feeding protocol:* Continuous feeding with 1.5 kcal/mL Fresubin® (Fresenius Kabi, Egypt) in the form of 18 gm carbohydrates, 5.8 gm fat, and 5.6 gm protein per 100 mL, was administered through nasogastric tube, starting with a rate of 20 mL/h, the feeding rate was increased gradually till the target energy requirement was reached. According to the 2016 ASPEN/SCCM guidelines [3], for all patients, based on actual body weight, the target energy requirement was calculated as 25 kcal/kg/d and the protein requirement was 1.4 g/kg/day. The head of the bed was elevated to 35° to reduce aspiration risk, and GRV was checked every 4–6 h by USG.

Treatment was stopped if failure of feeding occurs. Therapy failure was defined as patients with two or more high GRVs (i.e., ≥ 250 ml) or who developed feeding intolerance symptoms, including vomiting, abdominal pain, regurgitation or abdominal distension, for 2 successive episodes even with a feeding rate ≤ 40 mL/h, or at the lowest rate. In these patients, enteral feeding was discontinued temporarily, and the study drug was stopped. Additionally, the study drug was discontinued by patients in case of being transferred from ICU, stopped tube feedings, or had a severe adverse reaction that was correlated to the study drug.

### Study procedures

All patients were subjected to the following*:*

#### Patient data collection

- Baseline characteristics: Demographic data of the participants; Age, gender, height, weight, Body mass index (BMI), Organ Function as assessed by the Sequential Organ Failure Assessment (SOFA), Severity of illness as assessed by the Acute Physiology and Chronic Health Evaluation II (APACHE II).—ICU admission date and diagnosis.
- Complete medication history, medical history as well as nutrition data were recorded for each patient.

#### Clinical assessment

- Physical Examination: Measurement of blood pressure, heart rate, temperature. Complete examination including cardiovascular, respiratory, and neurological examination. Abdominal examination was done focusing on signs of feeding intolerance including passage of stool, abdominal distension and rigidity, intestinal sounds, vomiting or gastroesophageal reflux.
- Nutritional Risk Assessment: Modified nutrition risk in critically ill (mNUTRIC) score was measured for each patient at baseline and at the end of the study.
- Cardiovascular Assessment: A 12-Lead ECG was done on each patient at the screening visit to exclude QT prolongation, and at the end of the study to detect any effect of Itopride or metoclopramide on the QT interval.
- Biochemical Investigations*:* Routine lab investigations for ICU: Complete blood count, liver function test, electrolytes, kidney function test, lipid profile and blood glucose were done at baseline of the study and at the end of therapy.

### Measurable outcomes

#### Primary outcome

Radiological Assessment of Gastric Emptying: Measurement of GRV in enterally fed critically ill patients is a convenient clinical tool that is widely used as a surrogate indication of gastric emptying, success of feeding, and possibly the risk of aspiration [20]. Gastric emptying was measured using 2-dimensional ultrasound, where it is usually characterized by measurement of changes in antral cross-sectional area (CSA) [21]. Residual gastric volume assessment was performed using a "GE LOGIQ E9" ultrasound device; all exams were done by the same physician who was a professional of the department of radiology. After 30 to 45 min from ingestion of the feed, participants were positioned in the right lateral decubitus position for 5 min, and then an echographic ultrasound examination was done to measure the CSA of the gastric antrum. The antral residues gastric volume was then calculated using a mathematical formula that was previously tested and validated (GRV (ml) = 27 + 14.6 × right-lat CSA-1.28 × age)). [22] This formula is accurate with a margin of error in measurements of only ± 6 ml between the predicted and measured volumes. [23] The time to perform the entire measurements did not exceed 5–10 min in any individual. GRV data were collected at three-time points; day 1, day 4 and day 7.

#### Secondary outcomes

1. Determining the adequacy of enteral nutrition & compliance with enteral nutrition orders was registered daily; EN volume ratio (VR) % considered as an index of efficacy of nutrient delivery, was calculated as follows:
  EN VR (%) = (administered volume of EN/ prescribed volume) × 100.
2. Adequacy of calories and protein (the total amount of energy or protein received from EN is divided by the amount prescribed and expressed as %) over the 7 days of therapy.
3. ICU Length of stay (LOS).
4. Occurrence of ADEs: If adverse events occurred, the time of onset, duration, severity, relationship to itopride or metoclopramide and the requirement of treatment were evaluated.
5. Incidence of infectious complications; Patients were assessed for pulmonary infection up to 3 days following the end of treatment in both groups.
6. Occurrence of vomiting and/or requirement of post-pyloric feeding tube insertion due to feed intolerance

## Data management and analysis

The data collected have been revised, coded, tabulated, and introduced to a PC using Statistical package for Social Science ((IBM Corp. Released 2011. IBM SPSS Statistics for Windows, Version 20.0. Armonk, NY: IBM Corp).

For Descriptive statistics: Data were checked for normality with shapiro wilk test and expressed as mean (standard deviation) for parametric numerical data or median (interquartile range) for non-parametric numerical data. For Non-numerical data, frequency and percentage were used. For Analytical statistics, the following tests were used, Student T Test, Mann Whitney Test, Chi-Square test and Paired t test. *P* < 0.05 was considered to be statistically significant.

Sample size was calculated using STATA program, setting the type-1 error (α) at 0.05 and lower at 80%. Result from previous study showed that the treatment with a prokinetic (erythromycin) produced a greater reduction in the GRV than another prokinetic (metoclopramide) (59 ± 4% versus 35 ± 6%, respectively) with 24% mean difference between the 2 medications [24]. Assuming a lower difference (5%) between Itopride and metoclopramide in GRV reduction, produced a sample size of 38 cases per group taking in account 25% drop rate.

## Results

**Fig. 1:**
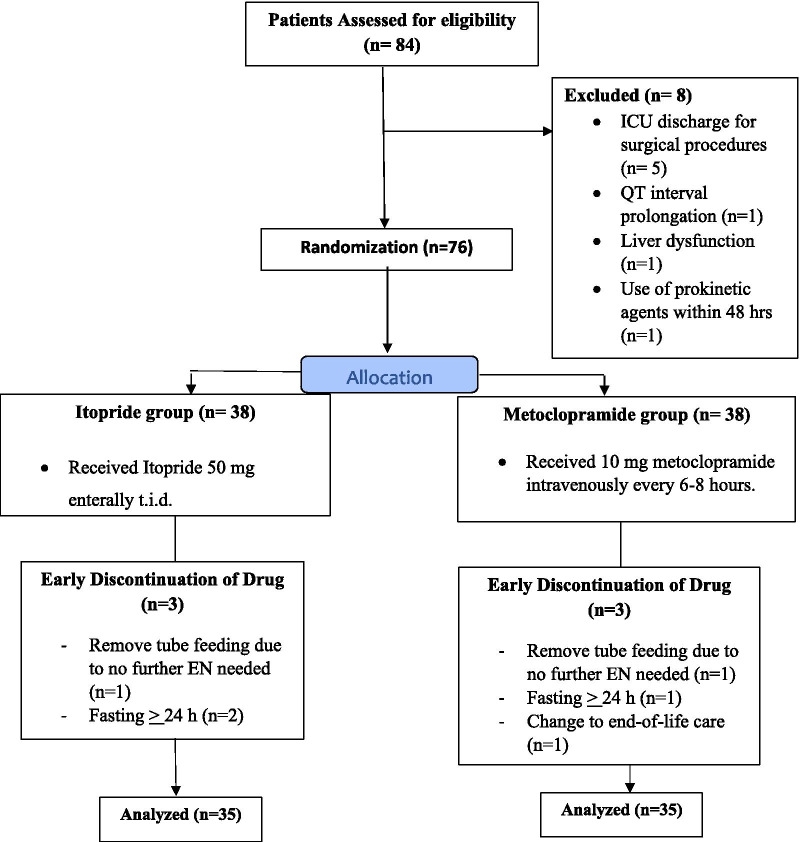
CONSORT flow chart diagram of study selection

Between October 2018 and November 2019, a total of eighty-four patients were screened, and finally 76 patients were randomly assigned to one of the 2 groups. The most frequent reason for exclusion prior to randomization were ICU discharge for surgical procedures. Thirty- eight patients were assigned Itopride and 38 received metoclopramide and due to dropouts, 35 patients of each group were finally analyzed (Fig. 1)

### Baseline characteristics

Table 1 shows that there were no statistically significant differences between the 2 groups in demographics, ICU admission diagnosis or risk scores (APACHE II, SOFA, mNUTRIC) at day 1 of the study.

**Table 1:** Baseline characteristics

	Itopride (n = 35)	Metoclopramide (n = 35)	*p*-value
**Demographics**			
Age, years, mean (SD)	43.94 (12.68)	42.09 (11.96)	0.53*
Height, m, mean (SD)	1.67 (0.11)	1.67 (0.09)	0.89*
Weight, kg, mean (SD)	65.94 (13.11)	67.09 (10.74)	0.69*
BMI, kg/m^2^, mean (SD)	23.41 (2.34)	23.91 (2.87)	0.42*
Male, n (%)	14 (40%)	18 (51.4%)	0.33**
Female, n (%)	21 (60%)	17 (48.6%)	
**Reasons for ICU admission**			
Surgical intervention, n (%)	10 (28.6%)	9 (25.7%)	
Trauma, n (%)	10 (28.6%)	7 (20%)	
CVS disorder, n (%)	6 (17.1%)	7 (20%)	
Respiratory disorder, n (%)	4 (11.4%)	6 (17.1%)	
Neurological disorder, n (%)	3 (8.6%)	3 (8.6%)	
Burn, n (%)	2 (5.7%)	3 (8.6%)	
**Risk scores, mean (SD)**			
APACHE II	22.19 (3.67)	21.79 (4.04)	0.66*
SOFA	8.60 (1.33)	8.83 (1.48)	0.49*
mNUTRIC	5.54 (1.22)	5.46 (0.98)	0.7*
**Enteral nutritional targets, mean (SD)**			
Volume prescribed (ml/day)	1310.66 (67.089)	1333.03 (63.461)	0.156*
Estimated energy requirements (kcal/d)	1757.14 (185.561)	1754.29 (137.932)	0.942*
Estimated protein requirements (g/d)	90.14 (8.357)	93 (6.207)	0.109*

*SD* standard deviation. *BMI* Body mass index. *APACHE II* Acute Physiology and Chronic Health Evaluation II. *SOFA* sequential organ failure assessment. *mNUTRIC* modified nutrition risk in critically ill

^*^Student *t* test

^**^Chi-Square Test

Differences between groups were not statistically significant (Wilcoxon signed rank test)

Additionally, at day 7 of the study, there were no differences between the Itopride and metoclopramide groups regarding APACHE II (*p* = 0.44), SOFA (*p* = 0.65) or mNUTRIC (*p* = 0.06).

There were no significant differences in the volume of feed, total energy or protein prescribed to patients between Itopride and metoclopramide groups (Table 1). Mean prescribed feed volume was 1310.66 ml (± 67.089) in Itopride group, and 1333.03 ml (± 63.461) in the metoclopramide group, (*p* = 0.156). Patients in the Itopride group were prescribed a mean of 1757.14 kcal/d (± 185.561) and the metoclopramide group 1754.29 kcal/d (± 137.932), (*p* = 0.942). Mean protein requirement in the Itopride group was 90.14 g/d (± 8.357) and 93 g/d (± 6.207) in the metoclopramide group, *p* = 0.109.

### Primary outcome

**Fig. 2:**
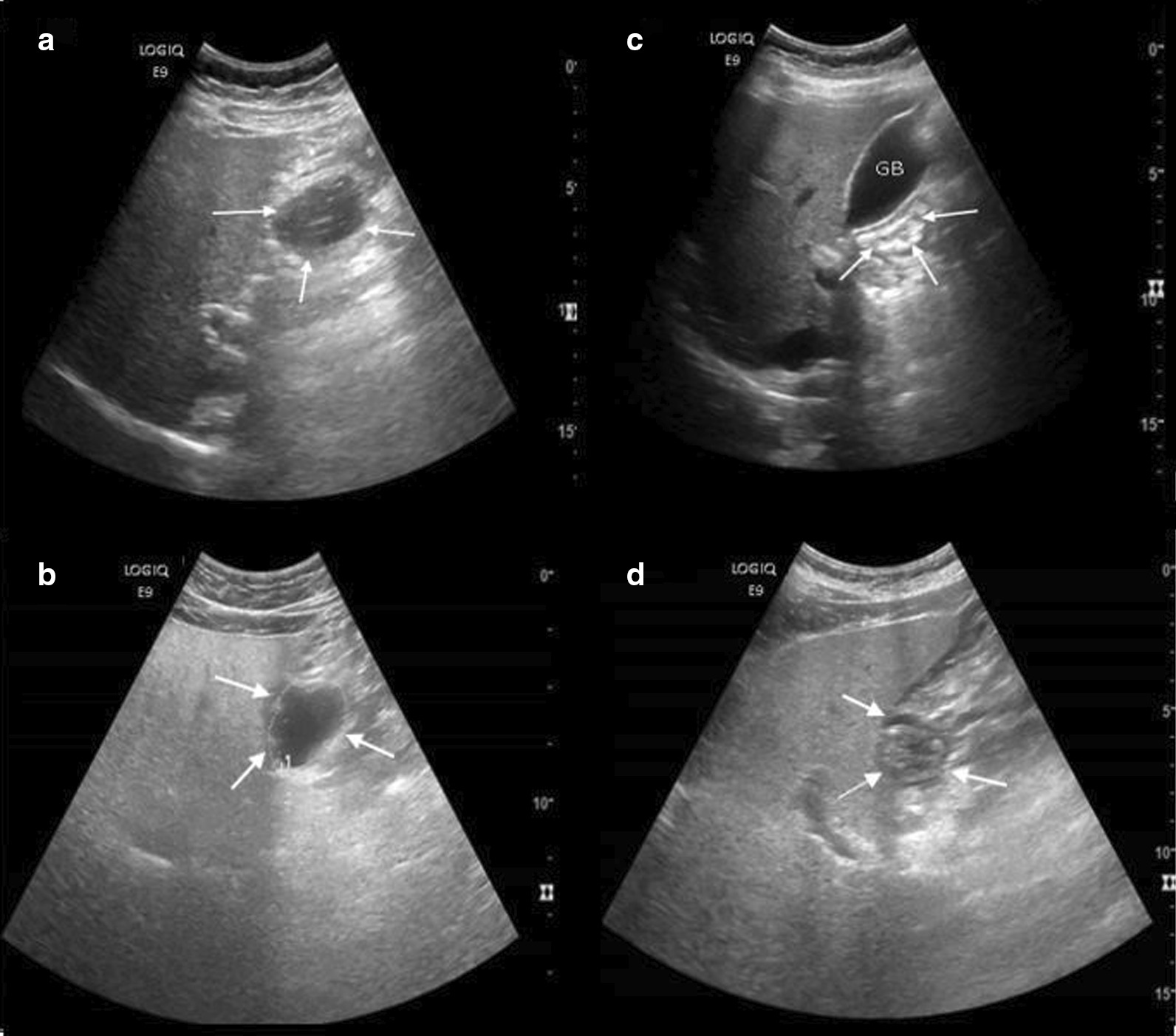
Ultrasound assessment of GRV. Cross Sectional Area of the gastric antrum (arrows) at high GRV (**a**, **b**) and low GRV (**c**, **d**) after ingestion of enteral feed. GB, gall bladder

**Fig. 3:**
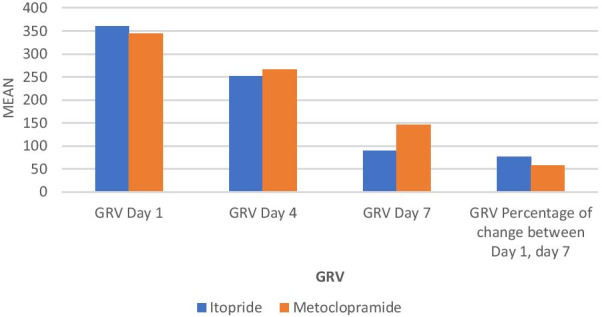
Effect of Itopride and Metoclopramide on GRV

The primary outcome was the radiological assessment of gastric emptying by measurement of GRV as shown in Fig. 2. In Table 2, at day 1, there was no statistically significant difference between the 2 groups regarding GRV with mean GRV of 359.5 (± 82.6) for the Itopride group and 344 (± 99.3) for the metoclopramide group, (*p* = 0.47). Similarly, at day 4, there were no differences between the mean GRV of Itopride 251.9 (± 77.6) and metoclopramide 265.9 (± 89), (*p* = 0.48). While, at day 7, GRV significantly decreased in the itopride group 89.6 (± 70.5) compared to metoclopramide group 145.8126 (± 66.7), (*p* = 0.001). Moreover, there was a statistically significant difference between the 2 groups regarding the GRV percentage of change between day 1 and day 7 with more decrease in GRV in the Itopride group 75.7 (± 18.8) than the metoclopramide group 57.3 (± 18.9) (*p* = 0.001) as shown in Fig. 3.

**Table 2:** Effects on Gastric residual volume

	Itopride (n = 35)	Metoclopramide (n = 35)	*p*-value
Day 1, GRV (mls), mean (SD)	359.5 (82.6)	344 (99.3)	0.47*
Day 4, GRV (mls), mean (SD)	251.9 (77.6)	265.9 (89)	0.48*
Day 7, GRV (mls), mean (SD)	89.6 (70.5)	145.8126 (66.7)	0.001*
Percent of change in GRV% between day1, day 7, mean (SD)	75.7 (18.8)	57.3 (18.9)	0.001*

GRV, Gastric residual volume

^*^Student *t* test

### Secondary outcomes

#### Difference in feed volume, energy and protein delivered between Itopride and metoclopramide groups.

**Fig. 4:**
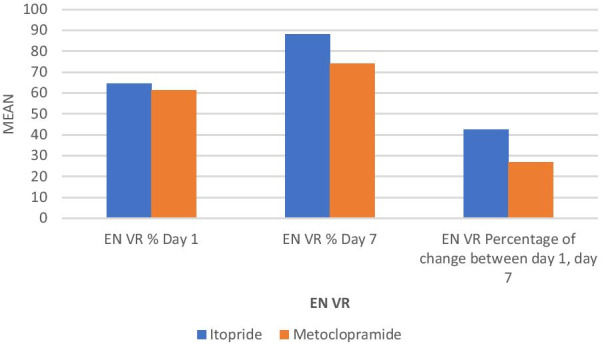
Effect of Itopride and Metoclopramide on EN VR

After the one-week therapy, mean prescribed feed volume delivered increased significantly in the Itopride group 1180.63 (± 63.42) than the metoclopramide group 1001.43 (± 70.955), *p* = 0.001. At day 1 of the study, there was no significant difference between the EN VR of Itopride 64.34 (± 14.20) and metoclopramide 61.2 (± 15.52), *p* = 0.364. Moreover, Fig. 4 shows that after the one-week therapy, the mean percentage of EN VR % was significantly higher in the Itopride 88.03 (± 9.32) than the metoclopramide group 74.17 (± 13.67), *p* = 0.001. And the percentage of change of EN VR between day 1 and day 7 was 42.32 (± 29.6) for the Itopride and 26.76 (± 36.51) for the metoclopramide, *p* = 0.003. Regarding the mean prescribed energy delivered, for the itopride group 1551.43 (± 216.417), it was significantly higher than the metoclopramide group 1390.14 (± 126.098), *p* = 0.001 and similarly, the percentage of energy ratio was higher in the Itopride group 89.158 (± 14.63) than the metoclopramide group 79.7 (± 9.397), *p* = 0.002. For the mean grams of protein reaching the patients of Itopride group 85 (± 8.135), it was significantly higher than the metoclopramide group 78 (± 8.419), *p* = 0.001. The ratio of protein administered was 94.83 (± 10.1) for the patients of Itopride group compared with 84.2 (± 10.26) for the metoclopramide group, *p* = 0.01.

#### ICU LOS

No statistically significant difference was found between both study groups concerning the ICU LOS (*p* = 0.71) as shown in Table 3.

**Table 3:** Secondary outcomes

	Itopride (n = 35)	Metoclopramide (n = 35)	*p-*value
Daily volume (ml), mean (SD)	1180.63 (63.42)	1001.43 (70.955)	0.001*
EN VR %, day 1, mean (SD)	64.34 (14.20)	61.2 (15.52)	0.364*
EN VR %, day 7, mean (SD)	88.03 (9.32)	74.17 (13.67)	0.001*
Percent of change in EN VR between day 1, day 7, mean (SD)	42.32 (29.6)	26.76 (36.51)	0.003**
Energy (Kcal/day), mean (SD)	1551.43 (216.417)	1390.14 (126.098)	0.001*
Energy Ratio %	89.158 (14.63)	79.7 (9.397)	0.002*
Protein (g/day), mean (SD)	85 (8.135)	78 (8.419)	0.001*
Protein Ratio %, mean (SD)	94.83 (10.1)	84.2 (10.26)	0.01*
ICU LOS (days), mean (SD)	15.97 (3.47)	16.23 (2.34)	0.71*

*EN VR* Entera nutrition volume ratio. *ICU LOS* ICU length of stay

^*^Student *t* test

^**^ Mann Whitney test

#### ADEs

**Fig. 5:**
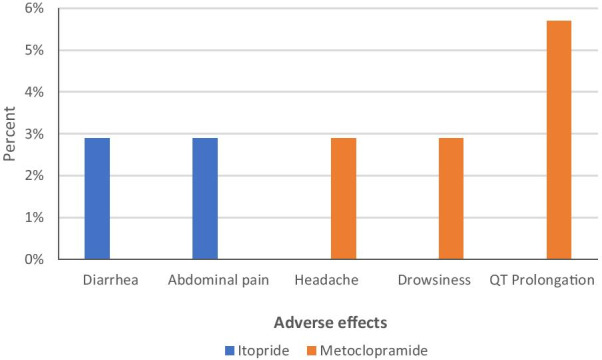
Summary of adverse effects

Adverse events were summarized in Fig. 5. Itopride was well tolerated and only minimal adverse events were documented; one patient suffered from diarrhea and one patient complained of abdominal pain. For the metoclopramide group, 1 patient suffered from headache and 1 patient suffered from drowsiness. All the reported adverse events from both groups were mild and subsided without interfering with continuation of treatment. At day 1, none of the patients of the 2 groups showed any prolongation of the QT interval. Therapy with Itopride was well tolerated and none of the patients showed any prolongation of QT interval on day 7 ECG. On the other hand, two patients of the metoclopramide group recorded QT interval prolongation.

Therapy with both drugs did not produce any abnormalities in serum biochemistry profile, where there were no significant differences between the 2 groups regarding all biochemical analyses at both day 1 and day 7.

Also, in patients of both groups, prolactin level was not increased and no adverse events like lacteal secretion occurred in both groups.

Neither vomiting nor requirement of post-pyloric feeding tube insertion due to feed intolerance or infectious complications were reported.

## Discussion

To the best of our knowledge, the current study is the first prospective, randomized, double-blind study to compare the effectiveness as well as the safety of Itopride against metoclopramide for EFI in critically ill patients.

Delayed GE causes major unwarranted clinical outcomes in critically ill patients with EFI and its rate is possibly underestimated as there are no clinically applicable GRV measurement techniques for these patients. Therefore, GRV monitoring is an essential component of EN patient care and aids in preventing complications due to EN. High GRV is a reliable surrogate marker of delayed gastric emptying, and GRV can still be useful for the early detection of delayed gastric emptying and the commencement of pharmacological treatment. Although GRV > 500 mL is identified as the threshold for the determination of large GRVs and according to the recent American recommendations, requires withholding of enteral feeding. [3] however, a GRV of 250 mL is defined as the threshold for the early detection of feeding intolerance and prompt initiation of therapy. Similarly, to our study, several other studies used ≥ 250 mL as an indication of high GRV [6, 24–26].

### Primary outcome

Regarding the primary efficacy outcome, the results of the present work demonstrate that Itopride has a significant effect in reducing the GRV after the one-week therapy duration while metoclopramide has a lesser effect, and this was also supported by the significant difference between the two groups in the percentage of change of GRV between day 1 and day 7. This finding may be explained that although the mechanism of action of itopride on GE is not clear so far, but the dual mechanism of itopride seems to make this drug efficacious in promoting the GI movement, where its mechanism of action is completely different from the existing prokinetics; it works both by inhibiting the activity of acetylcholinesterase and antagonizing the dopamine receptors, leading to enhancement of the cholinergic activity in the GI tract, which may in turn improve GI motility [14, 15].

Similarly, several studies have proven its efficacy causing symptomatic relief with minimal side effects compared to other prokinetic agents like demeperidone, cisapride and mosapride in other gastrointestinal disorders as functional dyspepsia and gastroesophageal reflux disease, where a meta-analysis studying nine well designed randomized placebo-controlled trials involving a total of 2,620 individuals of which 1,372 were treated with itopride at the dose of 50 mg t.i.d. each and 1,248 constituted the control group, who were treated with drugs such as domperidone, mosapride, or placebo. The effect of therapy in the group treated with itopride was significantly higher when compared with the control group, where individuals in the itopride group reported statistically significant improvement in GI dysmotility symptoms as post-prandial fullness, early satiation and global patient assessment scores compared to control group [27].

Moreover, two randomized controlled trials have reported that Itopride has been observed to have a positive effect on GE and gastroduodenal motility [15, 28]. Additionally, to support our results, a retrograde study in chronic gastritis Japanese patients where 50 mg itopride or placebo were administered reported that itopride accelerated GE [29]. In the same concern, another randomized, comparative study reported moderate to complete relief of gastrointestinal symptoms in 100% of non-ulcer dyspepsia patients treated with itopride as compared to 53% patients treated with metoclopramide [30]. Also, results from studies conducted among Indian patient population reported itopride as an efficacious drug in the management of GI dysmotility disorders [31, 32]. Likewise, a post marketing surveillance study carried out among 573 delayed GE patients reported global efficacy of itopride as excellent (44.5%), good (40.14%), fair (13.61%) and poor (1.75%) [33].

Furthermore, in experimental animal studies, itopride increased motility in the stomach, duodenum, and the proximal and distal colon [14, 34]. In addition, another study was carried out comparing the efficacy of Itopride and metoclopramide on rat's gastric motility and has shown the superiority of Itopride to metoclopramide in accelerating both upper and lower GI motility [35].

On the other hand, the decreased efficacy of metoclopramide at the end of the therapy may be explained that sometimes, tachyphylaxis related to the use of metoclopramide happens after a few days of therapy. Similarly, tachyphylaxis has been attributed to the use of metoclopramide as reported by other studies [13, 36]. Desensitization, downregulation and endocytosis of neurohumoral receptors have been proposed as mechanisms underlying the occurrence of tachyphylaxis [24].

Likewise, to the results of our study, a recent RCT reported that intravenous metoclopramide caused significantly higher accumulative GRV than another prokinetic (erythromycin estolate) combined with metoclopramide when used for EFI in critically ill patients [25].

### Secondary outcomes

Regarding the secondary outcomes, EN VR %, energy ratio%, and protein ratio % which indicate the adequacy of EN & compliance with EN orders.

It was observed that at day 7, the percentage EN VR, energy ratio and protein ratios were significantly higher in the itopride group than the metoclopramide group. This confirms the positive association between the decreased GRV (i.e., enhanced feed tolerance) in the itopride group with increased enteral feed intake. In the same concern, Mentec et al. reported that increased GRVs in their patients were correlated with reduced mean caloric intake [5]. This increase in the volume, calories and protein reaching patients of Itopride group compared to metoclopramide group can be explained by the positive effect of Itopride on EFI and thus improving feeding tolerance and achieving increase in the administered EN as compared to the prescribed EN.

Also, the results of our study demonstrated that energy target achieved was more than 80% for the Itopride group (89%) which is considered as successful feeding specifically in high nutrition risk critically ill patients and this was reported to improve clinical outcomes. This came in accordance with the results of another study that defined successful feeding for achieving ≥ 80% of energy target, [24]and this was different from other studies [6, 37]. In the same concern, the current trial showed high feeding success rates that came in accordance with the results of the PROMOTE trial [38], and compared with other ICU studies, the rates of aspiration, vomiting or regurgitation, and pulmonary infection were low [39, 40].

In our study, nutrition risk and illness severity were high similar to results of a recent randomized controlled trial [25] while they were evidently higher than those in previous study [41].

### Safety

The safety profiles of Itopride and metoclopramide were assessed where this study showed that itopride was well tolerated by most of the patients and likewise this was proved by several studies that showed good tolerability and minimal adverse events occurring in patients who received Itopride [27, 42].

In the current study, in Itopride group, only two patients suffered from mild symptoms of diarrhea and abdominal pain that resolved spontaneously without interference. Likewise, another post marketing surveillance study reported that the most reported ADEs were diarrhea, headache, giddiness, constipation, and itching/rash and most of them were mild and not related to itopride therapy [33]. While a relatively frequent problem in patients receiving EN and prokinetic agents is watery diarrhea, it is important to note that in the current study none of the diarrhea was correlated to infection, where microbiological testing of stool was negative for C. difficile toxin, inflammatory cells and bacterial infection in all patients.

Additionally, there were no reported CNS adverse events in the itopride group of the current study as Itopride is strongly polar, and thus, unlike other gastroprokinetic agents, hardly penetrates the brain and CNS. Therefore, CNS adverse effects and rises in serum levels of prolactin induced by itopride's antidopaminergic activity are less frequent and less severe than those associated with other dopamine receptor antagonists.

While, in the metoclopramide group, this study shows that two patients had adverse events of drowsiness and headache which may be attributed to the CNS ADEs caused by the drug. Likewise, previous study has reported CNS adverse events attributed to the use of metoclopramide [43].

In the present study also, we did not encounter any cardiac side effects with itopride. As Itopride does not cause prolongation of the QT interval, and, thus, unlike other prokinetics as metoclopramide and cisapride, does not likely cause arrhythmias.

However, since we have not included any patient with QT abnormality and with concomitant drug ingestion, further studies with itopride in high-risk groups would be needed.

On the other hand, after the one-week therapy, there were clinically related changes in the ECG of two patients of the metoclopramide group, particularly prolongation of QT intervals. In similarity to the findings of the current study, other studies and case reports have reported cardiotoxic effects caused by metoclopramide especially with higher doses and with long-term use [44, 45].

### GRV measurement by USG

Existing clinically applicable measurement options for GE include imprecise manual evaluation of GRV, the paracetamol absorption test limited by the availability of central laboratory and impaired by liver and renal dysfunction; or breath testing which has been limited by the need for cumbersome active and regular attachment/ detachment of the test tube to the patient. the wireless motility capsule test, GE breath test, magnetic resonance imaging, fluoroscopy, and paracetamol or synthetic glucose absorption. However, all these tests have technical limitations, are expensive, complex, have disadvantages such as long procedure time, invasiveness and radiation or not readily available in the ICU setting making the evaluation of GE a challenging task in clinical practice [17, 46, 47].

The current study showed that USG, which is a simple, non-invasive, widely available, inexpensive valid diagnostic test with a good inter-observer agreement, and which provides real-time structural and functional information regarding most parameters of gastric motility**,** can be easily used to measure GRV, potentially leading to improvement of patient management. It involves no radiation and can be performed at the bed side and the test does not involve radiation exposure and allows repeated measurements when the effects of drugs or therapeutic procedures are to be evaluated. Similarly, previous studies have reported the usefulness of two-dimensional US in assessing GRV and suggest that ultrasound accurately determines GRV [21, 48, 49].

Additionally, it was demonstrated to be comparable in sensitivity to scintigraphy which is considered the “gold standard” in evaluating GE [50].

This study has several strengths. First, the treatments were blinded and randomized. Second, this was the first study that demonstrated Itopride's superior efficacy to metoclopramide in reducing GRV and improved meeting of nutritional targets. Second, this study also demonstrated that measurements of GE can be performed by using a basic bed-side device which is USG that is readily available in any ICU and also in use in many medical applications and is validated for measuring GRV which gives credibility to the results obtained.

However, this study also has some limitations. First, the study was comparator, not placebo, controlled, it was also conducted at a single ICU; therefore, the generalizability of the results may be challenged, the study focused on a single center with its own feeding protocol; so, the findings of the study may not apply to other centers with different feeding protocols. In addition, the short duration of treatment and follow-up period to observe possible drug-related complications; thus, the frequency of complications may have been underestimated. Third, patients in this study were critically ill due to specific medical conditions; thus, the study findings may not be relevant to patients with critical illness caused by postoperative conditions or surgery. And although measuring GRV by USG was recommended by our study but it has some disadvantages which include the following: (1) the technique is dependent on the and needs expertise; (2) Bowel gas and/or obesity can restrict ultrasonic imaging; (3) geometric assumptions are needed for the shape of the stomach; (4) It is difficult to measure GE of solid meals using ultrasound [51, 52]. In addition, our study had high severity index and nutrition risk; so, the impact of therapy in patients with lower disease severity and nutrition risk could not be determined. Also, future studies using higher GRV thresholds are warranted.

Therefore, in order to optimize interpretation and generalizability, large, multicenter randomized controlled trials must be designed to further validate the safety and efficacy of Itopride in clinical settings and to confirm our results as Itopride is a promising prokinetic with high potency and safety compared to other prokinetics particularly metoclopramide. Our experience with USG suggests that further studies combining clinical assessment of EFI with GRV measurements may establish USG as a simple objective tool to guide individual prokinetic therapy.

## Conclusion

In summary, the findings of this study revealed that in the treatment of EFI, itopride, a dopamine D2 antagonist with anti-acetylcholinesterase effects, is superior to metoclopramide. The precise mechanisms by which itopride improves symptoms have yet to be determined, and more clinical studies are required to determine the effectiveness and optimal length of treatment in different populations.

Moreover, our study demonstrated that USG is a simple, non-invasive, inexpensive, and undemanding method for GRV measurements and can offer reliable assessments in the GE modality, where it can be used in other ICU settings rather than the other available methods with technical problems.

## Data Availability

The datasets used and analyzed during the current study are available from the corresponding author on reasonable request.

## Abbreviations

EN: Enteral nutrition
GI: Gastrointestinal
GRV: Gastric residual volume
ICU: Intensive care unit
EFI: Enteral feeding intolerance
ECG: Electrocardiogram
CNS: Central nervous system
ADEs: Adverse drug effects
GE: Gastric emptying
USG: Ultrasonography
CSA: Cross sectional area
VR: Volume ratio
BMI: Body mass index
SOFA: Sequential organ failure assessment
APACHE II: Acute Physiology and Chronic Health Evaluation II
mNUTRIC: Modified Nutrition Risk in Critically Ill
LOS: Length of stay
SD: Standard deviation
t.i.d.: Three times daily
GB: Gall bladder
